# Efficacy and complication of keyhole surgery and open surgery for repairing fibular collateral ligament in the persistent lateral ankle joint instability treatment: A protocol for systematic review and meta analysis

**DOI:** 10.1097/MD.0000000000039656

**Published:** 2024-09-13

**Authors:** Ding Wang, Jianhua Yuan, Yongping Wu

**Affiliations:** a Department of Orthopedics, The First People’s Hospital of Linping District, Hangzhou, China; b Department of Orthopedics, The Second Affiliated Hospital Zhejiang University School of Medicine, Hangzhou, China.

**Keywords:** ankle, instability, keyhole surgery, meta-analysis, open surgery

## Abstract

**Background:**

Over the past few years, there has been growing interest in identifying and treating persistent lateral ankle joint instability. Many medical professionals believe that early intervention is important to address this instability.

**Methods:**

Four databases on the outcomes of open and keyhole surgery for repairing the fibular collateral ligament in the treatment of chronic lateral ankle joint instability were acquired from the computer during controlled trials. Data analysis was conducted after the rigorous literature quality evaluation using Stata software.

**Results:**

This meta-analysis finally included 11 articles. Eleven studies reported complications were significantly lower in the treated group (odds ratio: 0.55; 95% confidence interval [CI]: 0.32–0.94; *P* < .05) compared to the untreated group, as were American Orthopedic Foot and Ankle Society scores (standard mean difference [SMD]: 3.77; 95% CI: 1.17–6.37; *P* < .01), visual analog scale scores (SMD: −0.69; 95% CI: −1.24 to −0.14; *P* < .05), Karlsson scores (SMD: 2.78; 95% CI: 0.35–5.21; *P* < .05), and Tegner scores (SMD: 0.41; 95% CI: −0.13 to 0.95; *P* = .139).

**Conclusion:**

According to complications, American Orthopedic Foot and Ankle Society scores, visual analog scale scores, and Karlsson scores, the study findings suggested that keyhole surgery may be beneficial in patients with persistent lateral ankle joint instability. Following this, the growing number of high-quality studies needs to confirm the results reported in this study.

## 1. Introduction

Ankle twisted sprain is a prominent injury in sports medicine.^[1,2]^ Larsen^[3]^ reported that approximately 30,000 people in the United States experience ankle sprains daily. Komenda and Ferkel^[4]^ observed that ankle sprains account for approximately 15% to 25% of orthopedic outpatient cases. The majority of ankle sprains can achieve complete recovery through conservative therapy, however, an estimated 15%−20% of people suffer from chronic ankle instability.^[4]^ Improper treatment can lead to recurrent symptoms and even cartilage damage, seriously affecting patients’ motor function and quality of life. Lateral ankle ligament injuries cause 80% of ankle ligament injuries. Studying the treatment plan for chronic lateral ankle instability is clinically significant because around 80% of ankle sprains present as injuries to the lateral ankle.

Over the past several years, there has been a growing focus on identifying and treating persistent side ankle instability. Many experts now agree that early surgical correction is essential for addressing this instability. Arthroscopy, when utilized in orthopedics and sports medicine, enables the identification and treatment of various degenerative changes resulting from chronic ankle instability. Through arthroscopic synovial clearance, free body removal, and cartilage repair, combined with lateral ligament repair and reconstruction, symptoms can be effectively alleviated, function can be restored, and ankle replacement and fusion can be delayed or avoided. Patients who have not responded well to conservative treatment have had surgical rejuvenation of the ankle joint fibular collateral ligament. This procedure aims to restore ankle stability and has shown positive clinical treatment outcomes. There are numerous surgical techniques documented both domestically and abroad, which can be broadly classified into 2 categories: anatomical rejuvenation and nonanatomical ligament rejuvenation. The modified Broström and the modified Karlsson procedures are both anatomical rejuvenation procedures that involve the tightening or reconstruction of the insertion point of the ligament, as well as the reconstruction of autologous or allogeneic tendons based on the original insertion point of the ligament. Nonanatomical rejuvenations like the Watson Jones, Evans, and Christman Snooks methods mainly involve stable reconstruction of the ankle joint in a forward and backward direction, with minimal impact on the lateral dynamics of the hindfoot. Furthermore, the function of the internal muscle group in the foot is unbalanced because of the lateral muscle group’s tendinous portion being used, weakening its tension. While the short-term impact is satisfactory, the long-term produces subtalar arthritis and increases stress during subtalar joint inversion movement.^[5]^ At present, anatomical reconstruction is the mainstream reconstruction scheme.

The most suitable technique for traditionally repairing the external collateral ligament is through Open Broström surgery. This method was initially suggested and implemented in clinical settings in 1980. The extensor support strap is used to suture the talofibular ligament in the anterior ankle and the calcaneus fibular ligament, repairing or strengthening them.^[6]^ Sports medicine professionals now acknowledge arthroscopic minimally invasive fibular collateral ligament repair because to the growing understanding of less invasive methods and the widespread use of arthroscopic technology. It is anticipated to take precedence over alternative choices when it comes to lateral ankle collateral ligament repair.^[7]^ As a minimally invasive surgery, it theoretically has advantages such as minimal trauma, mild postoperative pain, and accelerated recovery.^[8]^ Meanwhile, arthroscopy is used to repair the fibular collateral ligament and detect and treat any further abnormalities inside the joints. A comprehensive study was conducted to examine the impact of arthroscopic and open surgery on the repair of the lateral collateral ligament in the treatment of chronic lateral ankle joint instability.

## 2. Materials and methods

### 2.1. Studies selection

Type of study design results of open and arthroscopic surgery for lateral collateral ligament repair in the treatment of chronic lateral ankle joint instability are published in clinical trials. Preclinical trials, however, were not included.

### 2.2. Participants selection

Persistent instability of lateral ankle joint patients.

### 2.3. Types of interventions

Patients with chronic lateral ankle joint instability were treated with active arthroscopic care in the treated group, while patients in the control group underwent open surgery.

### 2.4. Types of resultant measures

Persistent lateral ankle joint instability patients: depending on the research, assessment tools for the effects of keyhole surgery and open surgery for fibular collateral ligament repairing in the persistent lateral ankle joint instability treatment are: complications; American Orthopedic Foot and Ankle Society (AOFAS) scores; VAS scores; Karlsson scores; Tegner scores. The literature evaluated resultant measures added to the research using at least any of the above scales.

### 2.5. Search approaches

The databases, including Cochrane Library, PubMed, Embase, and Web of Science, were accessed via the computer. The search terms are “Arthroscopic,” “Ankle Instability,” and “Open.” The time of the search was from the library establishment till July 2023. Certain steps for searching literature are related documents search in the English databases, title, abstract, and keywords reading, then search terms identified for the research work; search the database in English using “MeSH Terms” to recognize terms of subject, explored by a keywords and subject words combination.

### 2.6. Retrieval of data and qualitative assessment

First, an abstract was examined in detail. Following that, full-text reading was used to gather the results of the screening literature, which was carried out independently by 2 researchers. The screening findings were shared until a consensus was reached, and a third researcher examined the opposing literature. The data extracts basic information on the literature, research object, study type, sample size, intervention content, and subsequent measures.

### 2.7. Statistical analysis

Stata software was used to conduct this meta-analysis. Combined effects are: This study’s findings showed that a variety of assessment instruments are used, and all data are counted. There are variations among scores. Hence, standardized mean variation and 95% letters to the zone (confidence interval [CI]) are used as an effect indicator. Heterogeneity test: using Chi-square tests to determine heterogeneity among studies, if *P* > .1, *I*^2^ < 50%. The studies that were included reported heterogeneity. Fixed-effects model meta-analyses were conducted. If *P* < .1, *I*^2^ ≥ 50%, the analysis proceeds. The included studies demonstrated heterogeneity. Analyses were sources of a diverse nature. In meta-analyses, a random-effects model is employed when there is a deficiency of clinical heterogeneity.

## 3. Results

### 3.1. Search outcomes

**Figure 1.**
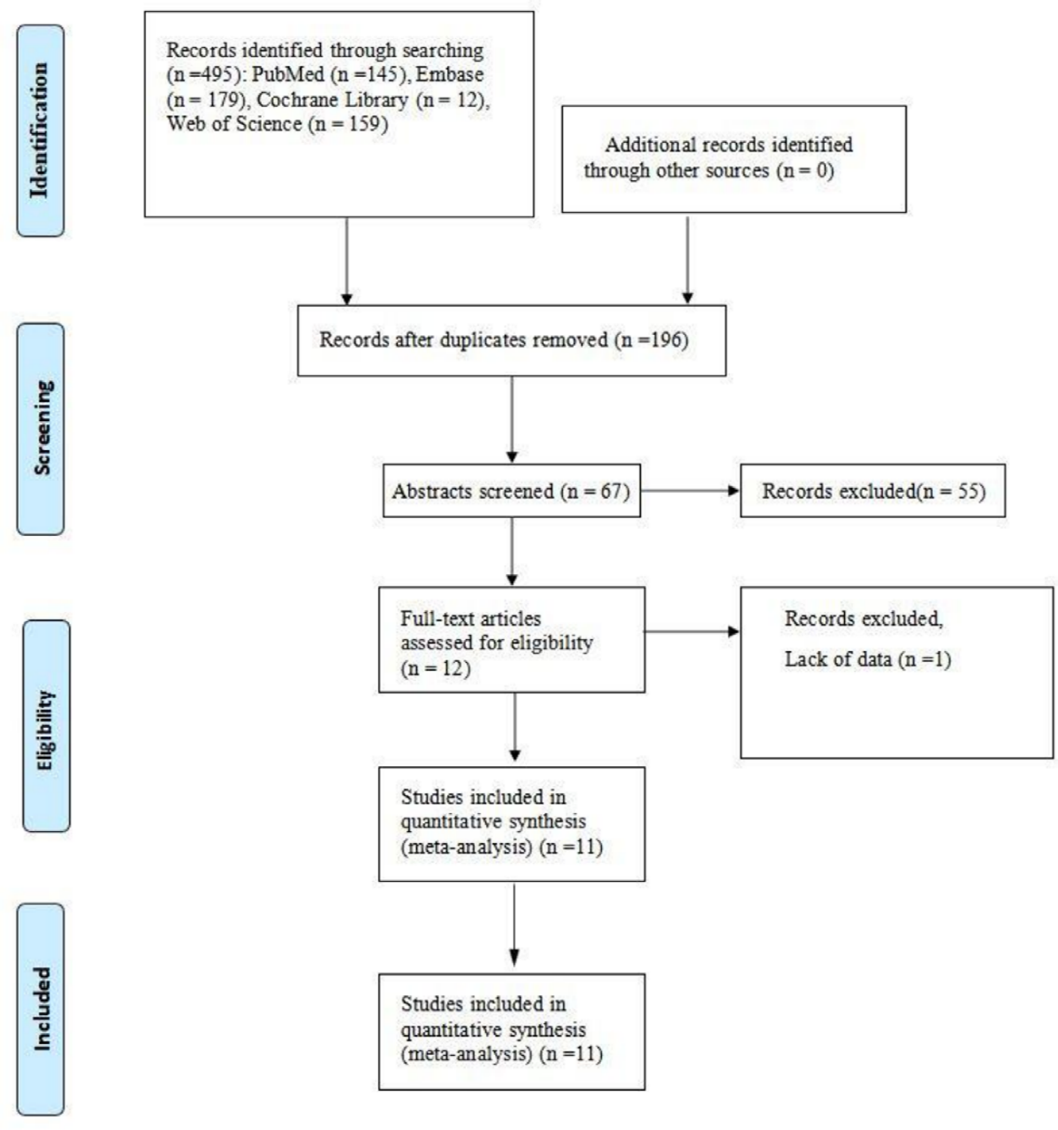
Flowchart.

Based on search strategies, 495 references were found. Following the exclusion of duplicate studies, 67 studies were reviewed based on their abstracts and titles. Furthermore, the whole text contained assessments of 12 articles. Then assessment of the entire text, 1 record was exempted for the following reasons: no data (n = 1). Eventually, 11 studies^[9–19]^ were added to the systemic review (Table 1). This process is shown in the PRISMA statement flowchart (Fig. 1).

**Table 1:** The basic characteristics of the included studies.

Study (ref.)	Sample size (T/C)	Male/female	Age (yr) (Mean ± SD) (T/C)	T	C	Main outcomes
Zeng et al^[9]^	17/10	22/5	30.9 ± 6.0/27.7 ± 9.7	AR	OS	①②④
Woo et al^[10]^	26/26	32/20	33.4 ± 10.6/31.5 ± 10.3	AR	OS	②③
Xu et al^[11]^	32/35	49/18	33.7 ± 7.0/35.8 ± 8.5	AR	OS	②③④⑤
DeVries et al^[12]^	43/12	22/33	44.7 ± 13.2/39.5 ± 16.0	AR	OS	①
Su et al^[13]^	31/26	35/22	30.6 ± 11.4/29.5 ± 9.1	AR	OS	①②③④⑤
Yeo et al^[14]^	25/23	19/29	35.2 ± 11.8/34.3 ± 14.1	AR	OS	②③④
Matsui et al^[15]^	19/18	20/17	28 (8–59)/24 (13–56)	AR	OS	①
Li et al^[16]^	23/37	47/13	30.3 ± 10.1/28.7 ± 8.7	AR	OS	①②⑤
Yang et al^[17]^	10/10	8/12	37.3/35.4	AR	OS	①②③
Mederake et al^[18]^	14/11	3/22	36.5 (17–53)/34 (17–54)	AR	OS	①
Hou et al^[19]^	36/34	34/36	28.3 ± 5.4/28.6 ± 4.8	AR	OS	①②③

① = complications, ② = AOFAS scores, ③ = VAS scores, ④ = Karlsson scores, ⑤ = tegner scores, AOFAS = American Orthopedic Foot and Ankle Society, AR = arthroscopy, C = control group, OS = open surgery, T = trial group, VAS = visual analog scale.

### 3.2. Complications

**Figure 2.**
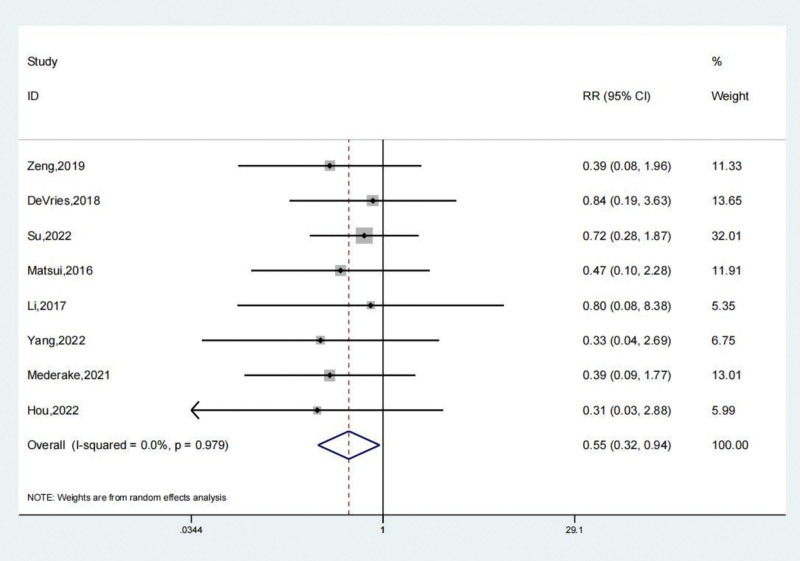
Forest illustration of the complications.

**Figure 3.**
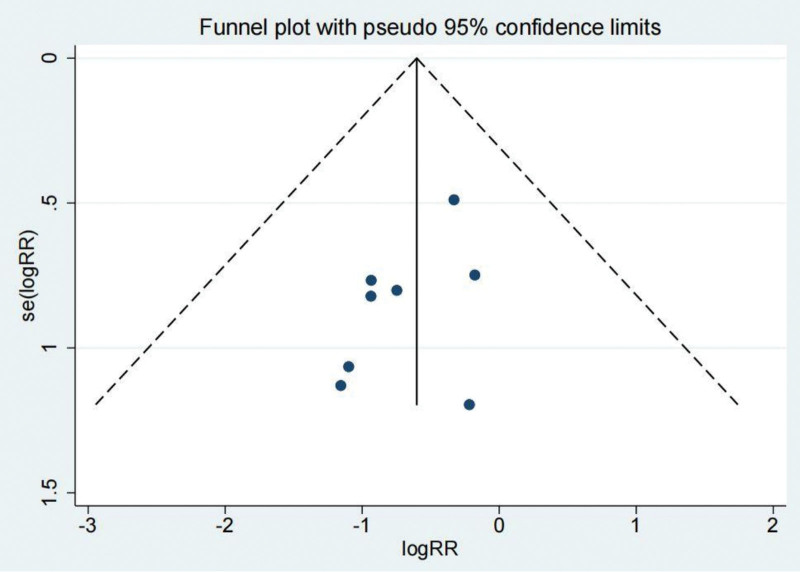
Funnel diagram of the complications.

**Figure 4.**
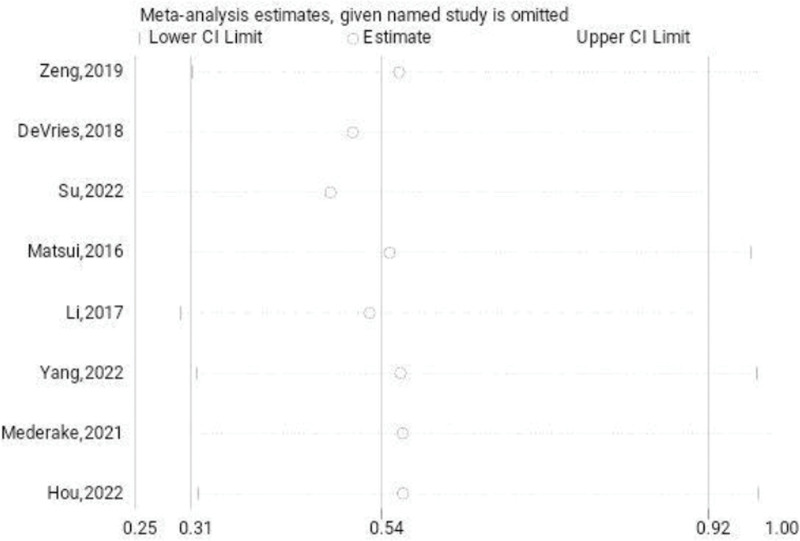
Sensitivity analysis of the complications.

Eight investigations documented the complications experienced by both the treated and untreated groups. A systematic analysis revealed that the incidence of complications in the treatment group was significantly lower (odds ratio [OR]: 0.55; 95% CI: 0.32–0.94; *P* < .05, Fig. 2) compared to the untreated group. Figure 3 depicts a funnel diagram that shows a notable degree of symmetry. The trial results revealed low heterogeneity and a sensitivity analysis was carried out (Fig. 4). When performed to treat patients with chronic lateral ankle joint instability, arthroscopic surgery decreases the number of complications compared to the untreated group. The Begg test yielded a value of 0.108, while the Egger test yielded a value of 0.171. These results indicate that the study findings were relatively uniform, and there is no clear evidence of publication bias.

### 3.3. AOFAS scores

**Figure 5.**
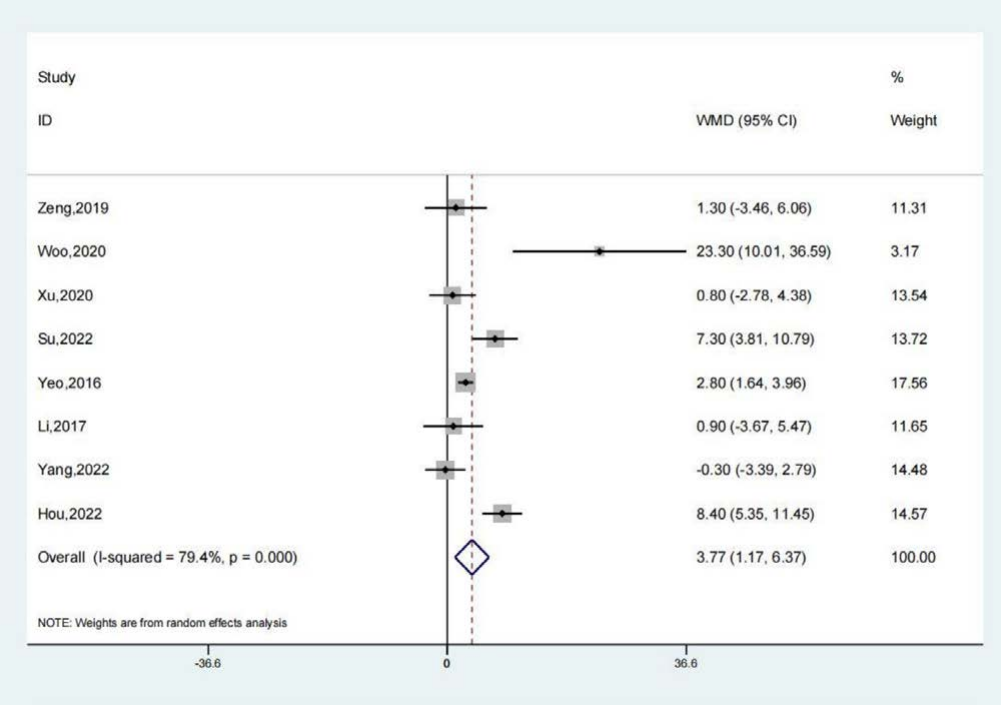
Forest illustration of the AOFAS scores. AOFAS = American Orthopedic Foot and Ankle Society.

**Figure 6.**
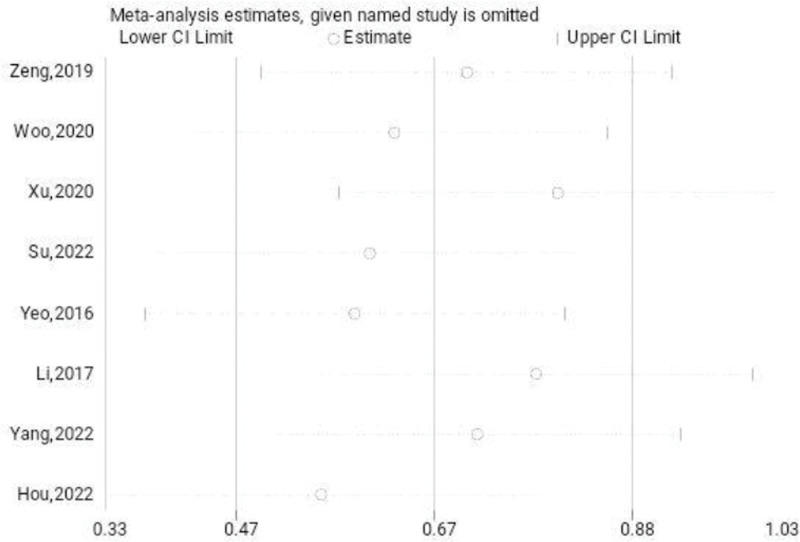
Sensitivity analysis of the AOFAS scores. AOFAS = American Orthopedic Foot and Ankle Society.

The AOFAS scores of the treated and untreated groups were suggested by the reports of 8 studies. The results of the systematic review showed that the treatment group’s AOFAS scores were significantly higher than those of the untreated group (SMD: 3.77; 95% CI: 1.17–6.37; *P* < .01, Fig. 5). The trial results showed significant heterogeneity, prompting a sensitivity analysis to be conducted (Fig. 6). Arthroscopic surgery, when utilized to treat patients with persistent lateral ankle joint instability, results in an increase in the AOFAS scores level, as compared to the group that did not receive any treatment. The Begg test yielded a value of 0.536, while the Egger test yielded a value of 0.468. These results indicate a very consistent outcome, suggesting a lack of evident publication bias.

### 3.4. VAS scores

**Figure 7.**
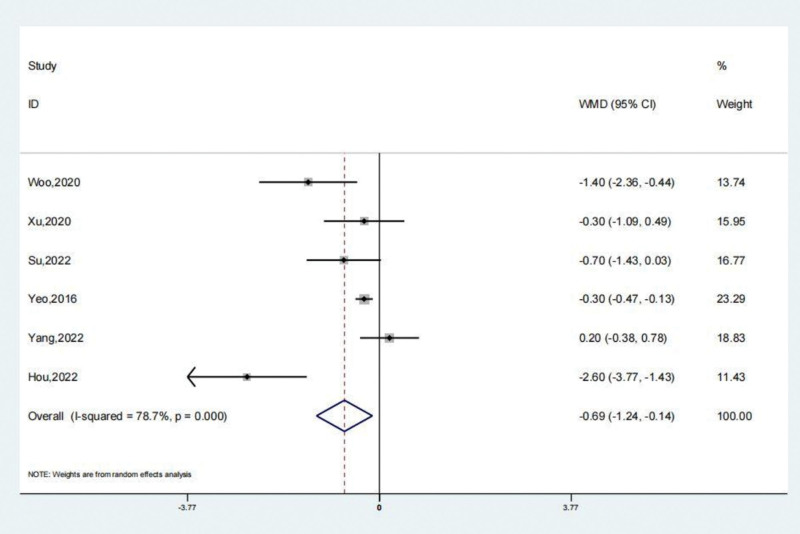
Forest illustration of the VAS scores. VAS = visual analog scale.

**Figure 8.**
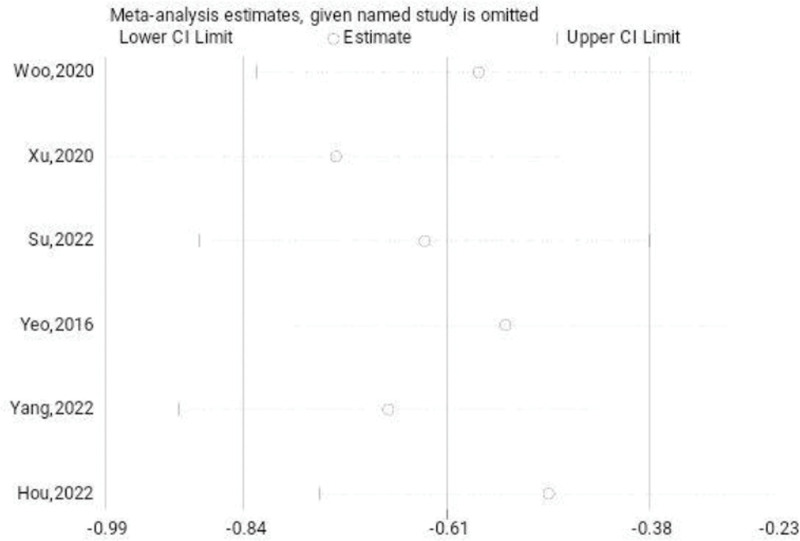
Sensitivity analysis of the VAS scores. VAS = visual analog scale.

The reports of 6 studies suggested that VAS scores of the treatment and the untreated group. A systematic review revealed that the VAS scores of the treatment group were remarkably lesser (SMD: −0.69; 95% CI: −1.24 to −0.14; *P* < .05, Fig. 7) compared to the untreated group. The trial results showed significant heterogeneity, prompting a sensitivity analysis to be conducted (Fig. 8). Arthroscopic surgery, when employed to address patients with persistent lateral ankle joint instability, effectively decreases the level of VAS scores compared to the untreated group. The Begg test yielded a value of 0.060, while the Egger test yielded a value of 0.254. These results indicate a consistent pattern and suggest a lack of significant publication bias.

### 3.5. Karlsson scores

**Figure 9.**
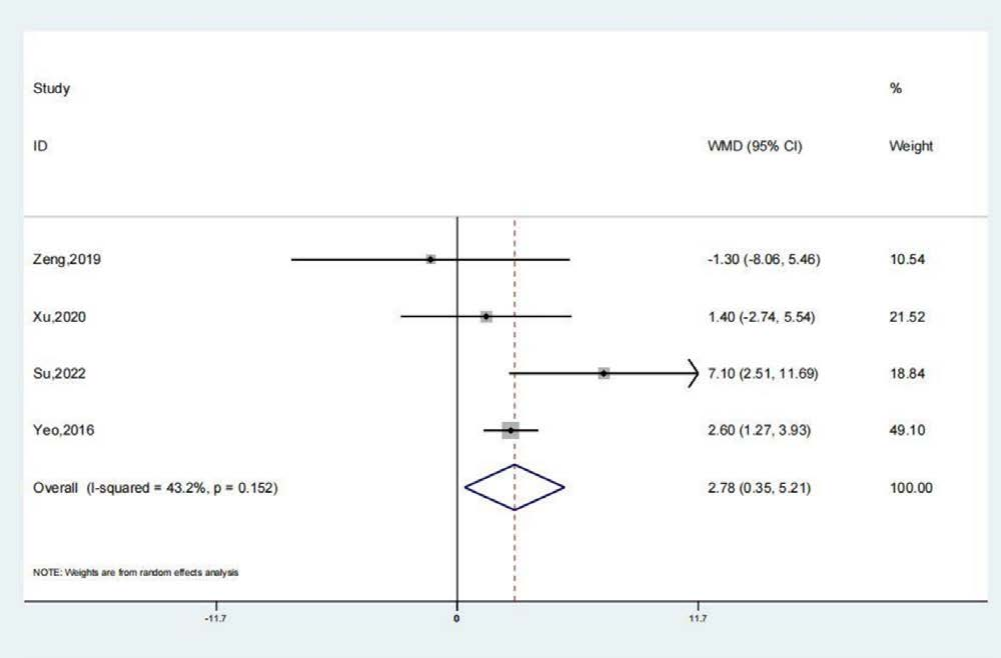
Forest illustration of the Karlsson scores.

The reports of 4 studies suggested that Karlsson scores of the treatment and the untreated group. A systematic review revealed that the Karlsson scores of the treatment group were remarkably greater (SMD: 2.78; 95% CI: 0.35–5.21; *P* < .05, Fig. 9) compared to the untreated group. Arthroscopic surgery, when utilized to treat patients with persistent lateral ankle joint instability, resulted in an increase in the Karlsson scores level, as compared to the group that failed to receive any treatment. The Begg test yielded a value of 0.734, while the Egger test yielded a value of 0.999. These results indicate a high level of consistency in the research findings and suggest that there is no significant publication bias present.

### 3.6. Tegner scores

**Figure 10.**
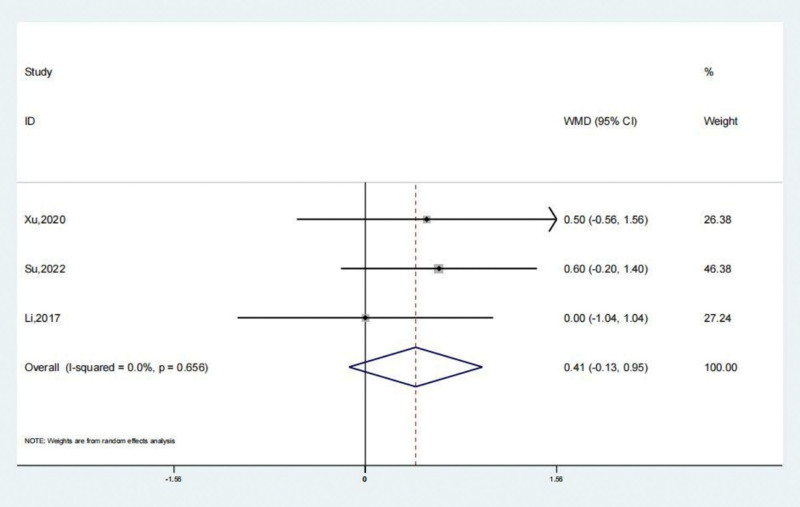
Forest illustration of the Tegner scores.

The reports of 3 studies suggested that Tegner scores of the treatment and the untreated group. A systematic review revealed that the treatment group’s Tegner scores lacked remarkable statistical significance (SMD: 0.41; 95% CI: −0.13 to 0.95; *P* = .139, Fig. 10) compared to the untreated group. Arthroscopic surgery, when employed to address persistent lateral ankle joint instability patients, did not result in an elevation of Tegner scores level, unlike the untreated group. Begg test yielded a value of 1.000, while Egger test yielded a value of 0.543. These results indicate a very consistent outcome and suggest a lack of evident publication bias.

## 4. Discussion

Orthopedic surgeons have determined that when an ankle injury causes a fracture, internal or external fixation should be applied as necessary, along with anatomical or functional reduction of the fracture. Regarding the management of ankle joint problems involving ligament damage, there are still different opinions. Furthermore, patients may suffer from lifelong problems because of inadequate treatment. Many patients and doctors consider it a minor injury for a superficial external ankle ligament injury, especially in the acute phase. Nevertheless, despite a reduction of pain, edema, and other symptoms following basic treatment methods like rest and pain management, certain clinicians disregard this injury, leading to inadequate and inaccurate therapy, which in turn exposes patients with chronic pain, reduced physical activity capacity, and limited range of movement. Hence, it is imperative to conduct further research and comprehend the influence of acute lateral ligament injury in the ankle on motor function. Current research shows that the incidence of simple injury or damage in the anterior talofibular ligament in ankle joint plantar flexion inversion injury is 66%; an incidence of concomitant injury to the calcaneal and fibular ligaments is 20%. Autopsy studies confirmed that the anterior talofibular ligament exhibits the lowest maximal load and energy absorption when it is fractured compared to the sideward ligaments. Specifically, it is 2/5 of the load-bearing capacity of the calcaneal ligament and 1/2 of the weight-bearing capacity of the posterior talofibular ligament. Therefore, the anterior talofibular ligament is the most susceptible to injury, while injuries to the calcaneal or posterior talofibular ligament are uncommon. An acute lateral ligament ankle joint injury can be readily mistaken for a simple sprain and ignored, resulting in the development of persistent instability, with probability ranging from 10% to 30%. Chronic ankle joint instability over a prolonged period can lead to traumatic arthritis in the tibiotalar and subtalar joints. This condition is characterized by symptoms including swelling, pain, and limited mobility, which severely restrict patients’ daily activities and motor abilities. Ultimately, patients may require ankle fusion or replacement, resulting in significant pain and financial burden.

The ankle joint lateral complex includes the calcaneal fibular ligament, anterior talofibular ligament, and posterior talofibular ligament. The component that thickens the joint capsule from the outer ankle tip to the front of the talus body is called the anterior talofibular ligament. Its main purpose at the ankle joint’s plantar flexion position is to restrict varus. The anterior talofibular ligament is the most susceptible side ligament of the ankle joint and the strongest when subjected to varus stress.^[1,20]^ There are many surgical methods for treating chronic ankle instability, including repairing direct ligament and reconstructing ligament.^[21,22]^ The Broström–Gould treatment, which entails directly repairing and suturing the anterior talofibular ligament and afterward suturing the extensor retinaculum to the outside of the ankle joint to further strengthen the lateral ankle stability, is currently the most commonly performed traditional operation.^[23]^ When performing Broström–Gould surgery alone, it is challenging to detect intra-articular lesions. Particularly treating the broken lateral ankle ligament, we also need to address intra-articular complications in cases with intra-articular lesions. Therefore, there is a specific practical benefit to combining ligament suturing with arthroscopy. It could both identify intra-articular lesions and manage intra-articular outcomes.

Over the past 5 years, there has been a gradual increase in clinical data on the use of keyhole reconstruction techniques for the lateral ankle joint ligament complex. This has led to a more objective evaluation of joint function prognosis. Brown et al^[24]^ conducted a meta-analysis and included 4 controlled studies. One of the results was that the keyhole surgery group outperformed the open surgery group in terms of short-term AOFAS functional score.^[24]^ The findings indicate that individuals recover from arthroscopic surgery more quickly and easily after the procedure. There were 276 patients in the treatment group and 242 patients in the untreated group according to the study and 11 additional literatures. The comprehensive evaluation concluded that individuals with chronic lateral ankle joint instability who underwent arthroscopic surgery had a lower risk of complications compared to those who received no treatment. The systematic review found that the therapy group experienced satisfactory complications (OR: 0.55; 95% CI: 0.32–0.94; *P* < .05). Based on preliminary results of a systematic review, the AOFAS scores of the therapy group were significantly higher compared to the untreated group (SMD: 3.77; 95% CI: 1.17–6.37; *P* < .01). The results of the systematic evaluation of VAS scores showed that the treatment group’s VAS scores were significantly lower than those of the untreated group (SMD: −0.69; 95% CI: −1.24 to −0.14; *P* < .05). The systematic review revealed that the Karlsson scores of the treatment group were remarkably greater (SMD: 2.78; 95% CI: 0.35–5.21; *P* < .05). A systematic evaluation demonstrated that the Tegner scores of the treatment group did not show a statistically significant difference compared to the untreated group (SMD: 0.41; 95% CI: −0.13 to 0.95; *P* = .139).

## 5. Limitations

The limitations of this meta-analysis include the restriction to English literature, the exclusion of literature in other languages, and the possibility of incomplete research inclusion and selection bias. Therefore, the focus should be on certain findings from the meta-analysis.

## 6. Conclusion

The study findings suggest that arthroscopic surgery may be a viable treatment option for individuals with persistent lateral ankle joint instability. This is supported by evidence of positive outcomes in terms of complications, AOFAS scores, VAS scores, and Karlsson scores. However, further high-quality investigations are required to confirm these findings.

## Author contributions

**Conceptualization:** Ding Wang.

**Data curation:** Ding Wang, Yongping Wu.

**Formal analysis:** Ding Wang, Jianhua Yuan, Yongping Wu.

**Investigation:** Ding Wang, Jianhua Yuan, Yongping Wu.

**Methodology:** Ding Wang, Jianhua Yuan, Yongping Wu.

**Writing—review & editing:** Ding Wang.

**Writing—original draft:** Yongping Wu.
